# Fatal Mycotic Aortic Aneurysmal Rupture Induced by Urosepsis From a Vesico-Enteric Fistula and/or Acute Pancreatitis

**DOI:** 10.7759/cureus.30916

**Published:** 2022-10-31

**Authors:** Ken-ichi Muramatsu, Hiroki Nagasawa, Kouhei Ishikawa, Youichi Yanagawa

**Affiliations:** 1 Acute Critical Care Medicine, Juntendo University Shizuoka Hospital, Izunokuni, JPN

**Keywords:** sepsis, fecaluria, pancreatitis, vesico-enteric fistula, infectious abdominal aortic aneurysm

## Abstract

A 70-year-old unconscious man with hospital phobia was transported to our hospital. On arrival, he displayed consciousness disturbance, unstable circulation, and a hypothermic state. Based on the clinical symptoms, the results of whole body computed tomography (CT), and biochemical studies, he was diagnosed with urosepsis (induced by vesico-enteric fistula), hyperglycemic crisis, infectious abdominal aortic aneurysm (iAAA), gallbladder stone-induced pancreatitis, and multiple organ failure. He was treated with supportive therapy. The abdominal magnetic resonance image (MRI) revealed an abdominal aortic aneurysm (AAA) with an inflammatory aortic wall, paraaortic inflammatory lymph node swelling, and periaortic fat inflammation. His consciousness temporarily recovered, but he did not agree to undergo surgery. On day 28, he suddenly collapsed. We present a fatal case of iAAA induced by urosepsis from vesico-enteric fistula and/or acute pancreatitis, complicated by multiple organ failure. In aging societies, physicians should explore not only the cause of disease but also the severity of the pathology and define fatal complications in elderly patients.

## Introduction

A mycotic (infectious) aortic aneurysm (iAA), is a rare, life-threatening disease of the aorta for which the chance of an early diagnosis may be missed due to a lack of specific clinical, radiological, and laboratory features [1,2]. Infectious abdominal aortic aneurysms usually occur in the elderly, predominantly affecting immunocompromised patients, including patients with diabetes mellitus, liver cirrhosis, end-stage renal disease, alcoholism, chronic glucocorticoid therapy, post-transplantation immunosuppression, human immunodeficiency virus infection, drug abuse, and malignancy [1,2]. An iAA is an acute inflammatory response to pathogenic infection, which induces neutrophilic infiltration at the arterial wall. During this process, the collagenolytic and elastolytic enzymes are activated, concomitant with saccular lumen dilation leading to rupture. The mortality rate approaches 100% if the iAA is left untreated. We herein report a case of fatal iAAA rupture induced by urosepsis from vesico-enteric fistula.

## Case presentation

A 70-year-old man with hospital phobia who lived alone was found after a fall in his house when a neighbor visited him in the morning. The neighbor tried to call an ambulance, however, he refused it. When the neighbor revisited his house at noon, he was found in an unconscious state, so the neighbor called the ambulance. His personal and family history are not known.

On arrival, his vital signs were as follows: E3V2M5 on the Glasgow coma scale, blood pressure 73/40 mmHg, heart rate at 96 beats per minute with atrial fibrillation, respiratory rate at 23 breaths per minute, percutaneous oxygen saturation unmeasurable, and a body temperature of 29.5 °C. He was of medium build and height. No skin rashes or lesions were noted. His head was normocephalic and atraumatic. His pupils were 3 mm in diameter and reacted promptly to light stimulation. His face was symmetric. His head and neck were not tender and showed no thyromegaly or adenopathy. His neck was supple, with a full range of motion. He had a regular rate and rhythm of heartbeat, and his lungs were clear on auscultation. His abdomen was soft but not tender and had no bruits. His back was straight without any midline defect, and he showed no cyanosis, clubbing or edema in the extremities. There was no gross evidence of paresis, but strength and sensation were difficult to assess. No involuntary movements were noted. Reflexes were symmetric in each extremity.

**Figure 1:**
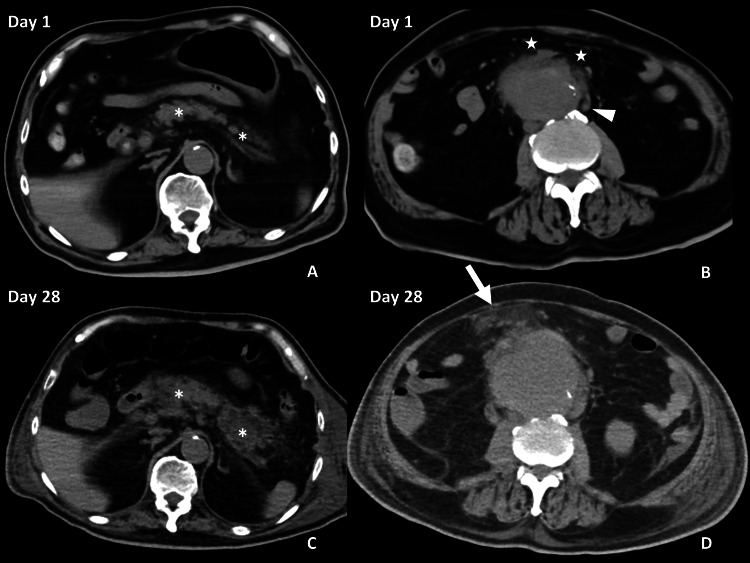
Plain abdominal CT on day one (A and B), and on day 28 (C and D) CT showed an abdominal aortic aneurysm (AAA) with an unclear margin (B, star) and paraspinal lymph node swelling (B, arrowhead) on arrival. The pancreas showed a normal appearance (A, asterisk). On day 28, the AAA expanded with the dirty fat sign observed on the ventral side (D, arrow). The pancreas became swollen (C, asterisk).

**Figure 2:**
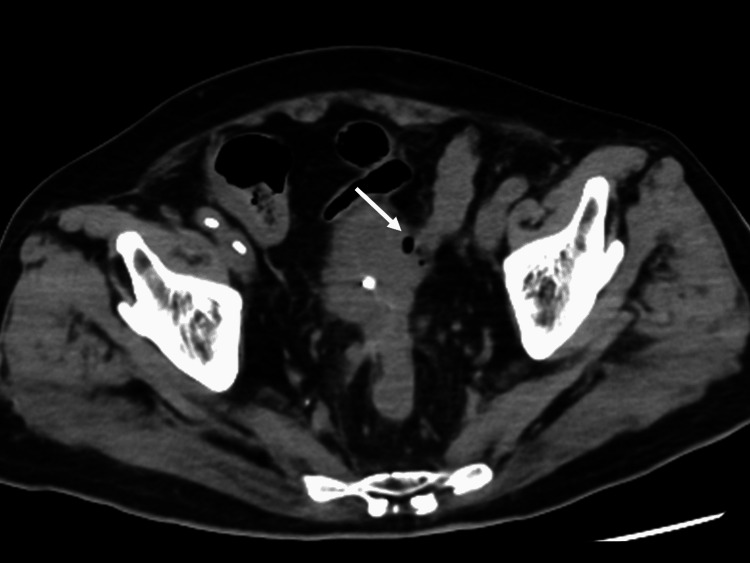
Plain pelvic CT on arrival Pelvic CT showed small amounts of gas at the bladder wall where it contacted the sigmoid colon.

A venous blood gas analysis revealed the following: potential of hydrogen (pH) 7.294, partial pressure of carbon dioxide (PaCO2) 23.0 mmHg, bicarbonate (HCO3) 10.8 mmol/L, base excess -13.9 mmol/L; lactate 3.3 mmol/L, glucose 1068 mg/dL. Whole body computed tomography (CT) showed gallbladder and common bile duct stones, iAAA due to unclear margin, the collapse of the inferior vena cava, and signs of vesico-enteric fistula that were initially overlooked (Figure 1 and Figure 2). His pancreas initially showed a normal appearance.

The blood test results of the patient are shown in Table 1.

**Table 1:** Blood test results HbA1C: Glycated hemoglobin

Variables	Level	Normal range
White blood cell count	11,700/mm^3^	3600-8900
Hemoglobin	15.4 g/dL	11.1-15.2
Platelet count	5.5 x 10^4^ /mm^3^	15.3-34.6×10^4^
Total protein	6.5 g/dL	6.5-8.5
Albumin	2.0 g/dL	4-5.2
Total bilirubin	0.9 mg/dL	0.4-1.2
Aspartate aminotransferase	32 IU/L	5-37
Alanine aminotransferase	20 U/L	6-43
Creatine phosphokinase	796 IU/L	47-200
Amylase	760 IU/L	43-124
Lipase	1131 IU/L	13～55
Blood urea nitrogen	185.2 mg/dL	9-21
Creatinine	3.69 mg/dL	0.5-0.8
Glucose	1050 mg/dL	65-109
HbA_1_C	11.30%	4.6-6.2
Sodium	138 mEq/L	135-145
Potassium	4.7 mEq/L	3.5-5.0
Chloride	96 mEq/L	96-107
Calcium	8.6 mg/dL	8.8-10.4
Ammonia	59 μg/dL	30-80
C-reactive protein	15.1 mg/dL	under 0.30
Activated partial thromboplastin time	35.3 seconds	30
Prothrombin time	41%	70-120
Fibrinogen	408 mg/dL	160-400
Fibrinogen degradation products	9.6 μg/mL	under 10

Urinalysis showed a urinary tract infection. Based on the clinical symptoms and results of radiological and biochemical studies, he was diagnosed with urosepsis (induced by vesico-enteric fistula), hyperglycemic crisis, iAAA, gallbladder, and common bile duct stone-induced pancreatitis, hypothermia, renal failure, coagulopathy and thrombocytopenia. Coma was attributed to shock, sepsis, and/or hyperosmolarity.

**Figure 3:**
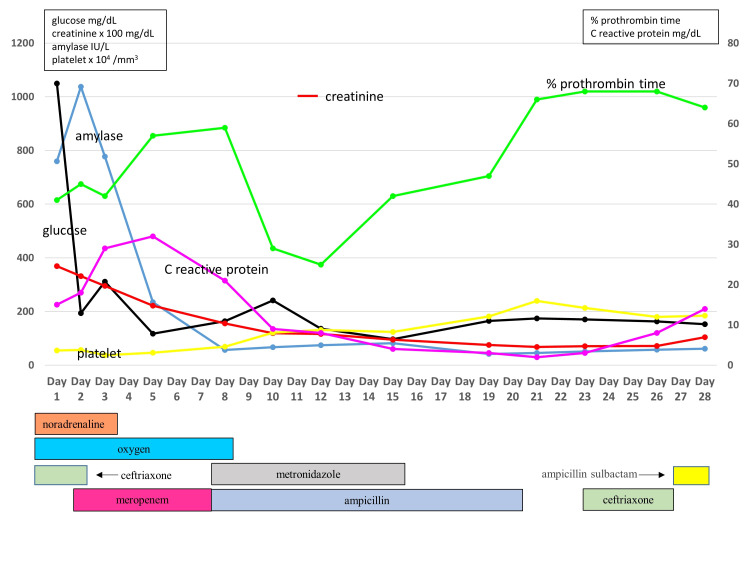
Clinical course during hospitalization The figure shows clinical course concerning change of laboratory results and main treatment.

He was treated with 1 g of ceftriaxone a day followed by 0.5 g of meropenem twice a day on day two of hospitalization due to deterioration of inflammation, massive infusion of lactate ringer fluid, continuous infusion of noradrenaline and regular insulin at 2 to 6 units per hour for 48 hours after bolus injection of 10 units of regular insulin, 5 L per minute of oxygen with a mask, and rewarming. His initial sequential organ failure assessment (SOFA) score was 12 points. His clinical course is shown in Figure 3. 

**Figure 4:**
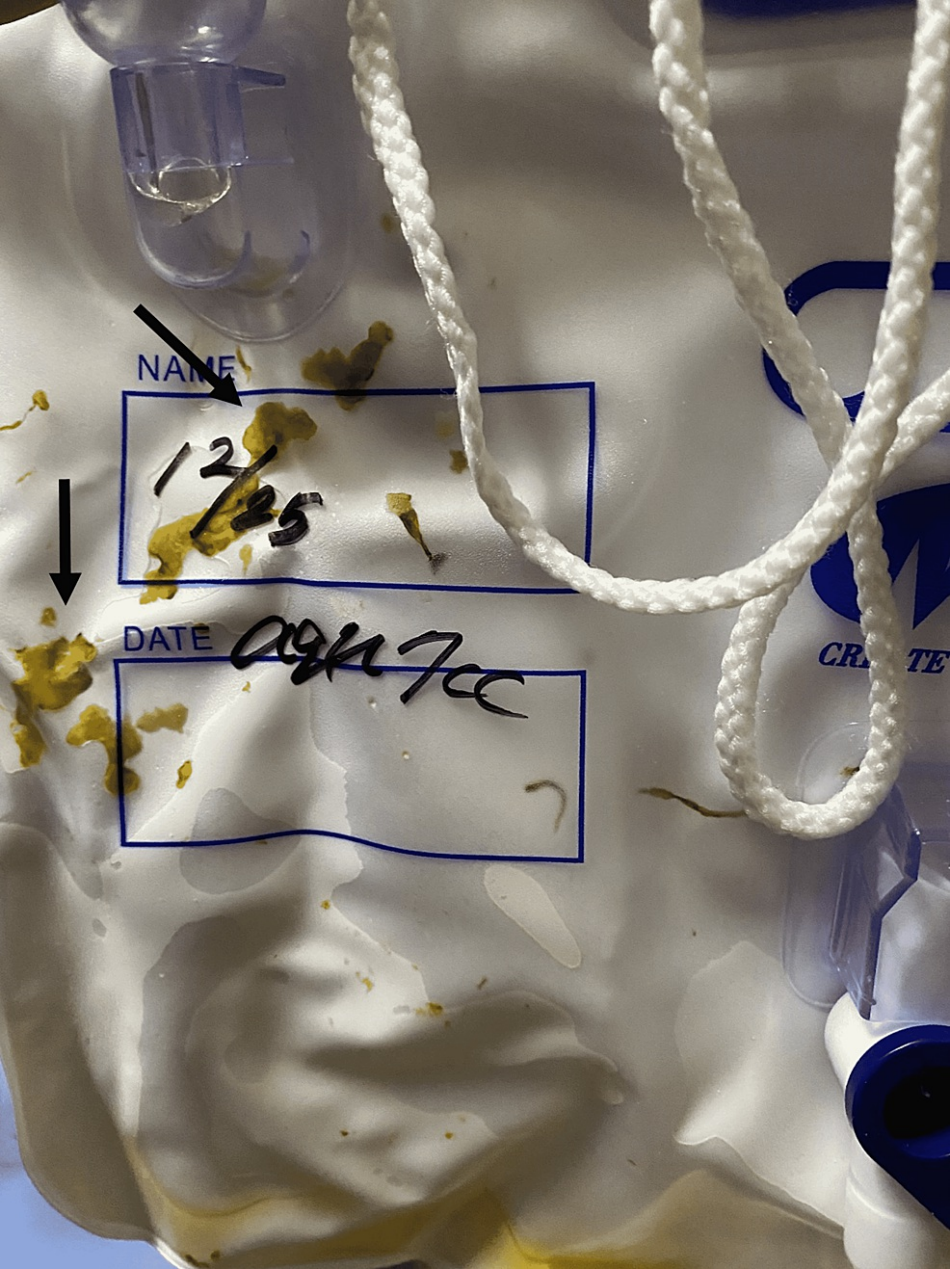
Macroscopic finding of urine The figure shows a stool-like substance (arrow) in a urine bag.

His next of kin did not agree to surgery for iAAA and vesico-enteric fistula; thus, he received supportive therapy. His urine bag showed a stool-like substance after admission; thus, a vesico-enteric fistula was suspected (Figure 4).

**Figure 5:**
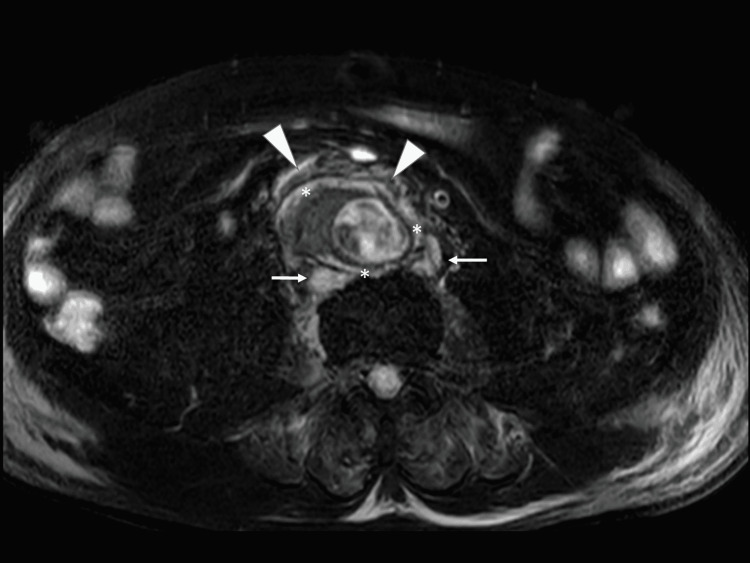
Abdominal short TI inversion-recovery MRI on day five The MRI showed a high abdominal aortic aneurysm with an inflammatory aortic wall (*), swelling of the paraaortic lymph nodes (arrow), and periaortic fat inflammation (triangle).

The patient's vital signs and multiple organ failure gradually improved after three days, so the massive infusion of lactate ringer fluid and continuous infusion of noradrenalin and regular insulin were all ceased. His systolic blood pressure was strictly controlled at <140 mmHg. Glucose intolerance was treated by intermittent injection of regular insulin depending on his serum glucose level. Abdominal magnetic resonance image (MRI) on day five revealed an AAA with inflammation of the aortic wall, swelling of the paraaortic lymph nodes and periaortic fat inflammation, which was compatible to iAAA (Figure 5) [2].

In addition, high-intensity signal change on the bladder wall where it contacted the sigmoid colon, was confirmed by MRI, which was compatible with vesico-enteric fistula.

*Escherichia coli*, *Lactococcus lactis* and* Actinomyces israelii*, were detected in urine cultures, while *Bacteroides fragilis* was detected in blood cultures. The diagnosis of vesico-enteric fistula was confirmed because no reports have described the detection of *Lactococcus lactis* in urine cultures. None of the bacteria showed drug resistance, and all had sensitivity to meropenem and ampicillin. As a result, meropenem was changed into 1 g of ampicillin three times a day and 0.5 g of metronidazole four times a day from day seven. His consciousness temporarily recovered (E4V4M6 on the Glasgow coma scale), and oxygen therapy was ceased on day eight, but he did not agree to receive radical treatment for iAAA and vesico-enteric fistula. The infusion of ampicillin was stopped on day 20, but he developed a fever on day 23 and received an infusion of ceftriaxone again. The results of a blood culture on day 23 were later found to be negative. On day 28, he complained of severe mid-abdominal pain having a SOFA score of 2 points on that day. Repeated CT showed that the iAAA had expanded with a dirty fat sign on the ventral side and ascites, in addition to fluid collection around the swollen pancreas (as seen above in Figure 1). He received pain and blood pressure control, as well as ampicillin/sulbactam. On the same day, he suddenly collapsed.

## Discussion

To our knowledge, this was the first reported case of fatal iAAA induced by urosepsis from a vesico-enteric fistula and/or acute pancreatitis complicated with multiple organ failure. Gallbladder stones, unstable circulation, and/or hypothermia in the present case may have played part in the development of acute pancreatitis [3,4]. Extremely rarely, acute pancreatitis co-exists with abdominal aortic aneurysm, which might result in rupture [5]. Prematurely activated pancreatic enzymes-mainly elastase-digest the elastic fibers of the tunica media, leading to disruption of the vessel wall or significant weakening of its structure, dissection, and aneurysm formation [5]. Accordingly, co-existent acute pancreatitis may facilitate the rupture of iAAA.

Based on the previous literature, iAAA was confirmed based on the results of the direct culture of the AAA wall [6]. Recently, cases tend to be treated by endovascular aortic repair using a stent, which is a feasible and effective method [7]. In such cases, the direct culture of the AAA wall is impossible. Instead, a radiological diagnosis becomes important, in addition to clinical signs (e.g., pain and/or fever). The iAAA appears on CT and MRI as a focal, contrast-enhanced, lobulated, saccular lumen, with an indistinct, irregular aortic wall. Gas bubbles that appear in and around the iAAA are highly reliable for diagnosing the iAAA. However, gas bubbles were not observed in the present case. Rapidly progressive growth of true or false aneurysms (>5 mm in two weeks), high signal intensity on T2-weighted MRI in the thickened iAAA wall, and/or edema of periaortic tissue and lymph nodes, suggest an infectious etiology similar to our findings. The iAAA in the present case was suspected from CT on arrival, however, the patient, who was treated conservatively, did not survive. There might be some criticism that the cause of death in the present case most likely was multiorgan failure secondary to severe sepsis and aging rather than due to a rupture of iAAA. However, until his sudden collapse, our patient had blood pressure high enough to require some manner of control, and his SOFA score was 2 points, so we believe that he ultimately died of rupture of iAAA.

Regarding vesico-enteric fistula, Kirsh et al. reported that the fistulas were most frequently attributable to diverticulitis, Crohn's disease, carcinoma of the colon, or other pelvic malignant conditions [8]. The present case may have been induced by diverticulitis, but this was not diagnosed. The most accurate diagnostic modalities were reported to be cystoscopy, cystography, and barium enema, while intravenous urography, intestinal endoscopy, and CT were less useful [8]. We also initially overlooked the signs of vesico-enteric fistula on the first CT scan. In the present case, fecaluria was the clue to the diagnosis [9]. In addition, the detection of* Lactococcus lactis *from the urine culture, which has not been reported previously, confirmed the diagnosis. If the present patient had agreed to surgical treatment, endovascular treatment using a stent for sudden fatal iAAA with the long-term use of antibiotics, followed by an open abdominal operation or closure of the fistula by clipping during colonoscopy (for the vesico-enteric fistula) and endoscopic stone extraction (for the gallbladder stone-induced pancreatitis) might have been the best treatments [7,9,10]. 

The patient seemed noncompliant with his medication and, as is common with neglected senior citizens, may have had psychiatric illnesses. His hospital phobia needed to be validated, as did his psychiatric illness status, however, we, unfortunately, did not conduct such investigations. In an aging society, multiple scenarios progress at the same time in elderly patients [11]. When treating such patients, physicians should explore not only the cause of the disease but also the severity of the pathology and define the fatal complications. In addition to standard examinations, whole-body CT might be useful for assisting in this management [12].

## Conclusions

We presented a fatal case of iAAA induced by urosepsis from a vesico-enteric fistula and/or acute pancreatitis, complicated with multiple organ failure. In this case report, we also presented a rare image of fecaluria. This fatal iAAA appeared on CT and MRI as a focal, contrast-enhanced, lobulated, saccular lumen, with an indistinct, irregular aortic wall. We emphasize that in an aging society, physicians should explore not only the cause of the disease but also the severity of the pathology and define fatal complications for elderly patients.
